# Hazard assessment of resin-based dental monomers: directions for greener dental material development

**DOI:** 10.2340/biid.v13.46213

**Published:** 2026-06-15

**Authors:** Mahmoud Abdelrahman Alatteili, Hamzeh ‘Ayed El-Amin’ Hassan Abid, Mohammad Talat Elsharkawy, Abdulmalik Saqer Mohammad Salti, Esraa Esam Mowafaq Shammamah, Rawan Yassin Nimir, Homa Darmani

**Affiliations:** Department of Biotechnology and Genetic Engineering, Faculty of Science and Arts, Jordan University of Science and Technology, Irbid, Jordan

**Keywords:** Dental resin monomers, bisphenol A, *Artemia salina*, developmental toxicity, metabolic effects, environmental safety

## Abstract

**Introduction:**

The increasing use of resin-based dental materials has prompted questions about their environmental footprint. During clinical procedures, small amounts of unpolymerized monomers may enter wastewater systems, and their possible effects on aquatic invertebrates remain insufficiently explored. We hypothesized that commonly used resin-based dental monomers could exert developmental and cellular effects in an aquatic invertebrate model.

**Materials and Methods:**

The acute and sublethal effects of five monomers associated with dental composites, namely – BPA (bisphenol A), Bis-DMA (bisphenol A dimethacrylate), Bis-GMA (bisphenol A-glycidyl methacrylate), Bis-EMA (bisphenol A ethoxylate dimethacrylate), and TEG-DMA (triethylene glycol dimethacrylate) – were evaluated in *Artemia salina* at both cyst and naupliar stages. The effects on naupliar survival, body length, and Lactate dehydrogenase (LDH) activity were measured to assess compound-specific biological responses.

**Results:**

Bis-GMA, Bis-DMA, and BPA significantly reduced survival, while Bis-EMA caused modest mortality, and TEG-DMA had no effect. Despite these differences in survival, most monomers affected naupliar growth, with greater developmental sensitivity observed following cyst-stage exposure. However, growth responses were compound specific and not uniformly dose-dependent across all monomers. LDH responses were monomer specific: Bis-GMA and Bis-DMA increased LDH activity, although Bis-DMA showed no consistent effect on growth, whereas BPA and TEG-DMA showed reductions in LDH activity, despite observed growth inhibition, and Bis-EMA showed no significant change.

**Conclusion:**

Taken together, our findings show that dental resin monomers differ in their developmental and metabolic impacts, highlighting the importance of considering safety alongside clinical performance when designing and selecting dental biomaterials. As the concentrations used were for hazard screening, these results indicate possible biological effects under controlled conditions but do not necessarily reflect environmental exposure scenarios or ecological risk. Additional studies at environmentally relevant exposure levels are needed to better evaluate ecological safety and support the development of more sustainable dental materials.

## Graphical abstract

**Figure:**
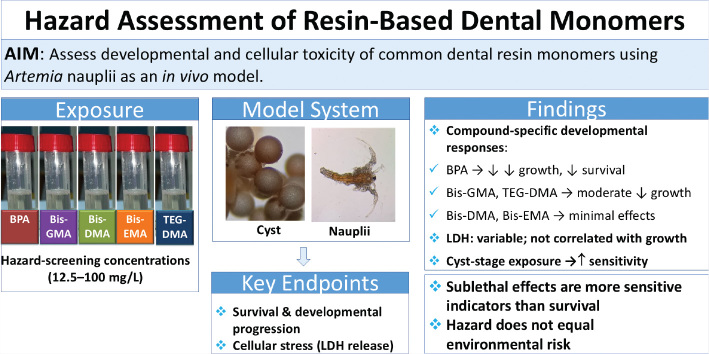
GRAPHICAL ABSTRACT

## Introduction

Emerging organic contaminants originating from industrial and consumer products are increasingly detected in aquatic environments, raising concern about their toxicological effects on nontarget organisms, particularly during early developmental stages. Among these contaminants, resin-related additives and monomers can enter surface waters through treated and untreated wastewater effluents, polymer degradation, and particle shedding. Once released, these compounds may be diluted, adsorbed onto sediments or organic matter, partially degraded, or persist depending on their physicochemical properties, all of which influence their environmental fate and bioavailability. Despite their widespread use, many of these compounds remain poorly characterized in terms of environmental toxicity.

Resin-based dental composites are among the most commonly used restorative materials in modern dentistry. While their clinical performance and biocompatibility are well documented, the elution of compounds from these materials and their potential environmental impacts are receiving increasing attention [1–6]. Dental composites consist of inorganic fillers dispersed within a resinous organic matrix synthesized primarily from methacrylate-based monomers [7, 8]. The principal monomer in most formulations is Bis-GMA (bisphenol A diglycidyl methacrylate), derived from BPA (bisphenol A), together with other dimethacrylate monomers, such as Bis-DMA (bisphenol A dimethacrylate), Bis-EMA (bisphenol A ethoxylate dimethacrylate), and TEG-DMA (triethylene glycol dimethacrylate), which is commonly added to reduce viscosity and improve handling [9–11].

Although resin-based composites are generally regarded as biocompatible in clinical settings, concerns remain regarding the leaching of BPA and BPA-derived monomers into the environment. BPA may be present as a residual contaminant or released through degradation of the resin matrix [12]. Both BPA and several methacrylate-based monomers exhibit estrogen-mimicking activity, raising concern about broader toxicological effects beyond human exposure scenarios [13]. During placement, finishing, removal, and long-term use of restorative materials, micro- and nanoscale particles containing unreacted monomers may be released into wastewater streams and subsequently reach aquatic ecosystems [1, 2, 4, 14, 15]. In aquatic environments, these compounds may undergo transport through wastewater treatment processes, dilution in receiving waters, or partitioning into sediments, which can influence organism exposure. Their persistence and resistance to biodegradation further underscore the importance of considering environmental fate in the safety assessment of dental materials.

While most toxicological studies on resin-based monomers have focused on human health outcomes, their effects on aquatic biota remain poorly characterized. *Artemia salina* (*A. salina*) is a widely used aquatic invertebrate model in ecotoxicology due to its suspension-feeding behavior, sensitivity during early developmental stages, and suitability for laboratory-based toxicity assays [16–18]. The *Artemia* lethality bioassay has been widely used as a low-cost and robust tool for assessing the acute and developmental toxicity of various organic contaminants, including dental-related materials [19–21]. However, toxicity data for individual resin monomers – particularly BPA and BPA-derived methacrylates – remain limited, especially for early life-stage exposure.

**Figure 1:**
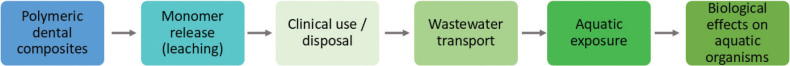
Conceptual framework illustrating the pathway of dental resin monomers from resin-based dental materials through release, environmental transport, aquatic exposure, and resulting biological effects in aquatic organisms.

Within this context, understanding how these compounds move from environmental release to biological exposure is important for interpreting laboratory findings in an ecological framework. A conceptual framework illustrating this source–fate–exposure–effect pathway is provided in Figure 1. Accordingly, the present study evaluated the acute and early developmental toxicity of five resin-associated monomers – BPA, Bis-GMA, Bis-EMA, Bis-DMA, and TEG-DMA – using *A. salina* as an established aquatic invertebrate model. By assessing survival and developmental endpoints at cyst and naupliar stages under controlled laboratory conditions, this study provides comparative information on the hazard potential of commonly used methacrylate-based monomers.

## Methods and materials

### Dental monomers

The methacrylate-based monomers tested include:

- BPA: Sigma-Aldrich (CAS 80-05-7);
- Bis-DMA: Acros Organics (CAS 3253-39-2);
- Bis-EMA (Plex 6878-0): Evonik (CAS 24448‑20‑2);
- Bis-GMA: Sigma-Aldrich (CAS 1565-94-2);
- TEG-DMA: Sigma-Aldrich (CAS109-16-0).

The monomers were dissolved in ethanol and diluted with artificial seawater (2.5% w/v sea salt). Control treatments comprised of artificial seawater supplemented with ethanol at 0.4% (v/v), corresponding to the maximum solvent level employed in exposure groups.

### A. salina cysts and hatching

Dormant *A. salina* encysted embryos (Carolina Biological Supply) were soaked in cooled distilled water (4°C, 1 h) and processed, as previously described [21]. Cysts that had sunk to the bottom were collected and placed in a container with artificial salt water at room temperature (22–25°C) under continuous illumination (yellow incandescent bulbs). Hatching occurred consistently within 48 h.

### Acute toxicity assay

The acute toxicity assay was conducted according to a previously published study [21]. After hatching, 20–35 nauplii were transferred into 3 mL of test solutions containing BPA, Bis-DMA, Bis-EMA, Bis-GMA, or TEG-DMA at 0, 12.5, 25, 50, or 100 mg/L in 12-well plates. The nauplii were incubated at ambient temperature for 24 h without feeding. After exposure, motionless (dead) nauplii were counted under a stereomicroscope. Total nauplii were enumerated following immobilization in 100% ethanol, and percentage viability was calculated. Each concentration was tested in triplicate wells per experiment, with three independent experimental runs. The well was treated as the experimental unit for statistical analysis.

### Effects of monomers on body lengths of A. salina nauplii

In a separate experiment, 9–12 nauplii were transferred into 3 mL of test solutions containing BPA, Bis-DMA, Bis-EMA, Bis-GMA, or TEG-DMA at 0, 12.5, 25, 50, or 100 mg/L in 12-well plates. After a 24-h exposure period, naupliar body length was determined from the microscopic image using VIS image analysis software. Multiple nauplii were measured within each well, and the mean body length per well was calculated. The well was treated as the experimental unit for statistical analysis. Each concentration was tested in three wells per experiment, with three independent experimental runs, yielding nine independent replicates per group.

### Exposure of A. salina cysts to monomers

To examine outcomes throughout early-stage growth, 20–30 soaked cysts were transferred into 3 mL of treatment solutions (0, 12.5, 25, 50, and 100 mg/L) in 12-well plates. After hatching, multiple nauplii within each well were measured for total body length as outlined above, and the mean value per well was calculated. The well was treated as the experimental unit for statistical analysis. Each concentration was tested in three wells per experiment, with three independent experimental runs, yielding nine independent replicates per group.

### Biochemical assays

For sublethal analysis, cysts were hatched in 40 mL of test solutions containing 12.5 mg/L of each monomer (BPA, Bis-DMA, Bis-EMA, Bis-GMA, or TEG-DMA). This concentration was chosen because it caused minimal mortality and no overt stress symptoms while allowing assessment of early sublethal cellular effects, including membrane integrity via LDH release. Controls were hatched in artificial seawater with an equivalent concentration of ethanol (0.4% v/v).

### Lactate dehydrogenase activity

Nauplii were washed with ice-cold PBS, weighed, and frozen at −80°C. Samples were lysed in Tris-HCl buffer (50 mM, 0.5% Triton X-100, pH 7.6; 200 μL/100 mg nauplii) containing protease inhibitors (Sigma-Aldrich®, Cat. No. S8830), homogenized using bead rupture (Omni Bead Ruptor 4; 8 cycles [5 m/s, 45 s] with 2-min intervals of cooling on ice), and centrifuged. Supernatants were collected for analysis.

LDH activity was quantified using a high-affinity LDH assay kit (Elabscience®, Cat. No. E-BC-K046-M) in 96-well plates, following the manufacturer’s protocol. Absorbance was read at 490 nm. Each treatment was performed in triplicate with appropriate blanks and controls.

### Protein quantification and normalization

Total protein content was determined using a BCA protein assay kit (SMART™, Cat. No. 21071). Samples (25 µL) were mixed with 200 µL of working reagent, incubated at 37°C for 30 min, cooled, and absorbance determined at 630 nm. LDH activity was corrected for total protein content and expressed as units per milligram protein.

### Statistical analysis

Naupliar survival was analyzed using binary logistic regression to evaluate the effect of monomer concentrations on viability, with concentration treated as a continuous predictor. Developmental effects of the monomers on naupliar body length were analyzed using one-way analysis of variance (ANOVA), followed by Tukey’s pairwise comparisons. One-way ANOVA was selected because the experimental design involved a single independent variable (treatment) with multiple independent groups, and no interaction effects were assessed due to the nonfactorial structure of the study. Data analyses were performed using Minitab version 17, and statistical significance was set at *p* < 0.05.

## Results

### Naupliar survival

**Figure 2:**
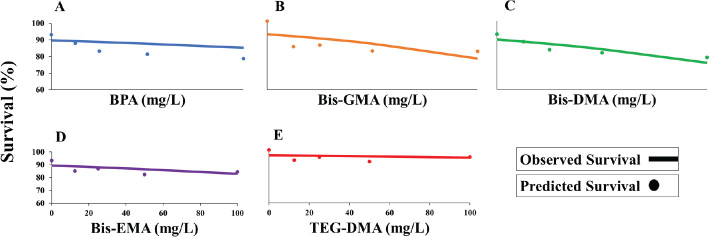
Naupliar survival of *A. salina* as a function of resin monomer concentration. Panels illustrate exposure to (A) BPA, (B) Bis-GMA, (C) Bis-DMA, (D) Bis-EMA, and (E) TEG-DMA. Symbols indicate observed survival proportions, and solid lines represent fitted logistic regression models describing concentration-dependent survival probability.

Naupliar survival declined with increasing concentrations of BPA, Bis-GMA, and Bis-DMA (Figure 2a–c). Logistic regression showed that increasing BPA concentration significantly reduced survival probability (χ² = 19.33, *p* < 0.001; odds ratio [OR] = 0.9897 per 1 mg/L increase, 95% confidence interval [CI]: 0.9853–0.9942). A similar concentration-dependent decrease was observed for Bis-GMA (χ² = 21.22, *p* < 0.001; OR = 0.9879, 95% CI: 0.9828–0.9929) and Bis-DMA (χ² = 17.81, *p* < 0.001; OR = 0.9902, 95% CI: 0.9857–0.9947).

Bis-EMA (Figure 2d) showed limited but statistically significant toxicity (χ² = 4.94, *p* = 0.026; OR = 0.9945, 95% CI: 0.9897–0.9993), whereas TEG-DMA (Figure 2e) had no significant effect on survival (χ² = 2.66, *p* = 0.103; OR = 0.9958, 95% CI: 0.9909–1.0008). Taken together, BPA, Bis-GMA, and Bis-DMA demonstrated clear concentration-dependent lethal effects; Bis-EMA showed weak toxicity and TEG-DMA did not significantly impair naupliar viability.

### Effects of resin monomers on A. salina nauplii body length

Because a single control group was shared across monomers and the design was not fully factorial, naupliar body length was analyzed using one-way ANOVA with treatment group as the fixed factor, followed by Tukey’s post hoc test.

### BPA

**Figure 3:**
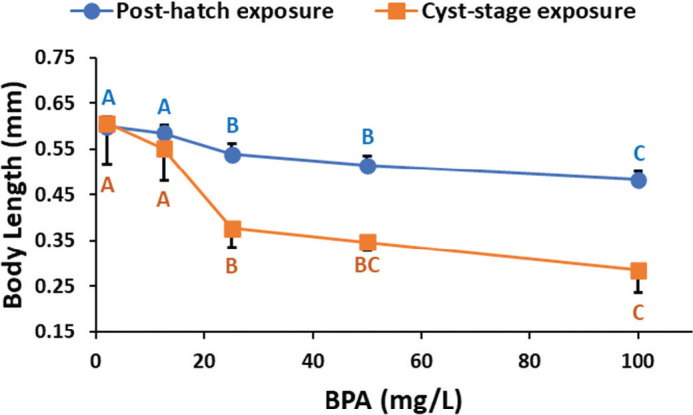
Effects of BPA concentration on naupliar body length of A. salina. Circles represent nauplii exposed after hatching, and squares represent nauplii hatched from cysts exposed to BPA. Data represent means ± SD. Means that do not share a letter are significantly different from each other (one-way ANOVA; Tukey’s pairwise comparisons).

Exposure of *A. salina* nauplii to BPA caused a strong, concentration-dependent reduction in body length (Figure 3) (*F*₄,₄₀₀ = 54.30, *p* < 0.001). Mean body length decreased from 0.6 mm in controls to 0.483 mm at the highest concentration (100 mg/L). Tukey’s post hoc test showed that 12.5 mg/L BPA was not significantly different from the control, while 25, 50, and 100 mg/L treatments resulted in significantly shorter nauplii. Moreover, 25 and 50 mg/L formed an intermediate group, and the highest concentration (100 mg/L) showed the greatest reduction in body length, significantly different from all lower concentrations. Relative to controls, mean body length was reduced by approximately 3%, 10%, 14%, and 19% at 12.5, 25, 50, and 100 mg/L, respectively.

### Bis-GMA

**Figure 4:**
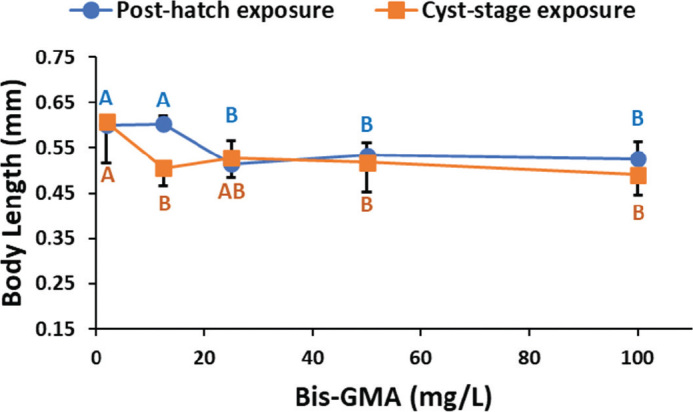
Effects of Bis-GMA concentration on naupliar body length of A. salina. Circles represent nauplii exposed after hatching, and squares represent nauplii hatched from cysts exposed to Bis-GMA. Data represent means ± SD. Means that do not share a letter are significantly different from each other (one-way ANOVA; Tukey’s pairwise comparisons).

Bis-GMA exposure significantly affected naupliar body length (*F*₄,₄₀ = 14.45, *p* < 0.001; Figure 4). Mean body length was similar between the control (0.600 mm) and the lowest concentration (12.5 mg/L; 0.603 mm), with no significant difference observed. In contrast, all higher concentrations (25, 50, and 100 mg/L) resulted in significantly reduced body lengths compared with the control and 12.5 mg/L treatment. However, no significant differences were detected among the 25, 50, and 100 mg/L groups, which formed a single homogeneous subset. Tukey’s pairwise comparisons. Relative to controls, naupliar body length was reduced by −1%, 14%, 11% and 12% at 12.5, 25, 50, and 100 mg/L, respectively.

### Bis-DMA

**Figure 5:**
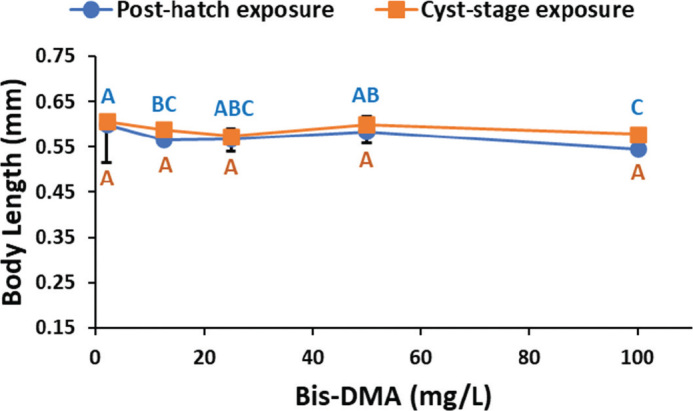
Effects of Bis-DMA concentration on naupliar body length of A. salina. Circles represent nauplii exposed after hatching, and squares represent nauplii hatched from cysts exposed to Bis-DMA. Data represent means ± SD. Means that do not share a letter are significantly different from each other (one-way ANOVA; Tukey’s pairwise comparisons).

Bis-DMA exposure showed a significant overall effect on naupliar length (*F*₄,₄₀ = 6.14, *p* < 0.005; Figure 5). Tukey’s pairwise comparisons revealed limited pairwise differences, with only the 12.5 and 100 mg/L exposure groups showing significantly shorter body lengths compared to the control, while the 25 and 50 mg/L groups did not differ significantly. Corresponding reductions in body length were 6%, 5%, 3%, and 9%, respectively, at 12.5, 25, 50, and 100 mg/L.

### Bis-EMA

**Figure 6:**
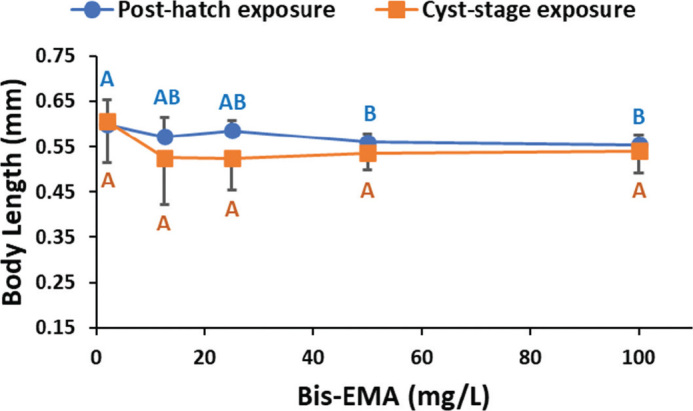
Effects of Bis-EMA concentration on naupliar body length of A. salina. Circles represent nauplii exposed after hatching, and squares represent nauplii hatched from cysts exposed to Bis-EMA. Data represent means ± SD. Means that do not share a letter are significantly different from each other (one-way ANOVA; Tukey’s pairwise comparisons).

Bis-EMA exposure had a significant overall effect on naupliar body length (F₄,₄₀ = 4.49, p < 0.005; Figure 6). Tukey’s pairwise comparisons showed that nauplii in the 50 and 100 mg/L groups were significantly shorter than those in the control group. The 12.5 and 25 mg/L groups exhibited intermediate body lengths and did not differ significantly from either the control or the higher-exposure groups. Compared with controls, body length was reduced by 5%, 2%, 7%, and 8% at 12.5, 25, 50, and 100 mg/L, respectively.

### TEG-DMA

**Figure 7:**
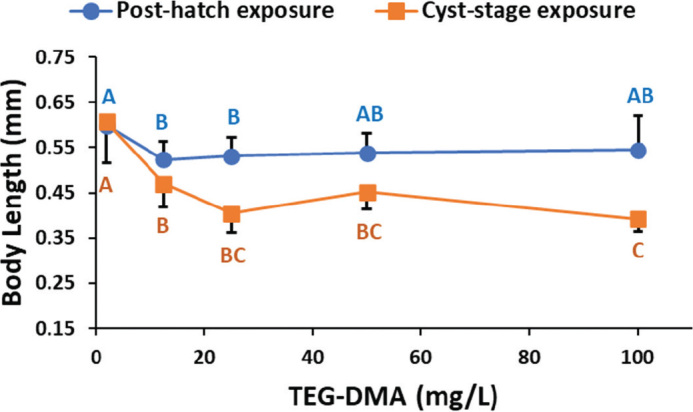
Effects of TEG-DMA concentration on naupliar body length of A. salina. Circles represent nauplii exposed after hatching, and squares represent nauplii hatched from cysts exposed to TEG-DMA. Data represent means ± SD. Means that do not share a letter are significantly different from each other (one-way ANOVA; Tukey’s pairwise comparisons).

TEG-DMA exposure caused a significant effect on naupliar body length (*F*₄,₄₀ = 3.68, *p* < 0.05; Figure 7). Tukey’s test indicated that the 12.5 and 25 mg/L groups were significantly shorter than the control, whereas the 50 and 100 mg/L groups were not significantly different from the control due to overlapping groupings. This resulted in a nonmonotonic response pattern across concentrations. Body length was reduced by 13%, 11%, 10%, and 9% at 12.5, 25, 50, and 100 mg/L, respectively.

### Body length of A. salina nauplii hatched from monomer-exposed cysts

Following exposure of *A. salina* cysts to the monomers, the hatched naupliar body lengths were quantified and analyzed by using one-way ANOVA, followed by Tukey’s post hoc test.

### BPA

*A. salina* nauplii hatched from cysts that had been exposed to BPA showed strong, dose-dependent reduction in naupliar body length (*F*₄,₄₀ = 48.57, *p* < 0.001; Figure 3). The average naupliar body length decreased from 0.61 mm in control nauplii to 0.28 mm, at the highest concentration (100 mg/L). Tukey’s post hoc test showed that no significant difference was observed between the control and 12.5 mg/L group. In contrast, the other BPA concentrations (25–100 mg/L) significantly reduced body length, relative to the controls. Nauplii from the 25 mg/mL group were significantly longer than those from the 100 mg/L group, while the 50 mg/L group was not significantly different from either the 100 mg/L groups. Relative to controls, body length was reduced by 9%, 38%, 43%, and 53% at 12.5, 25, 50, and 100 mg/L, respectively.

### Bis-GMA

Exposure to Bis-GMA caused a significant inhibition of naupliar development (*F*₄,₄₀ = 5.15, *p* < 0.005; Figure 4) compared with the control. However, no clear dose-dependent differences in body length were observed among the tested concentrations. Tukey’s post hoc test showed that control naupliar body length was not significantly different from the 25 mg/L group but were significantly shorter from the remaining treatments, which did not differ significantly from each other. Relative to controls, body length was reduced by 17%, 13%, 15%, and 19% at 12.5, 25, 50, and 100 mg/L, respectively.

### Bis-DMA

Bis-DMA exposure did not significantly affect naupliar length (*F*₄,₄₀ = 0.62, *p* = 0.652; Figure 5). Average body length did not differ among Bis-DMA concentrations and the control, as confirmed by Tukey’s test. Corresponding reductions in body length were 3%, 5%, 1%, and 5% at 12.5, 25, 50, and 100 mg/L, respectively.

### Bis-EMA

Bis-EMA exposure did not significantly affect nauplii body length (*F*₄,₄₀ = 1.93, *p* = 0.124; Figure 6) compared with the control. Tukey’s test confirmed that no significant differences were observed among any treatment groups. Corresponding reductions in body length were 13%, 14%, 12%, and 11% at 12.5, 25, 50, and 100 mg/L, respectively.

### TEG-DMA

TEG-DMA exposure during the cyst stage significantly reduced naupliar body length overall (*F*₄,₄₀ = 22.08, *p* < 0.001; Figure 7). All treatment groups were shorter than the control. Although the 100 mg/L group was significantly shorter than the other dose groups, mean body length did not decrease monotonically with increasing concentration (reductions of 22%, 33%, 25%, and 35% at 12.5, 25, 50, and 100 mg/L, respectively).

### LDH activity

**Figure 8:**
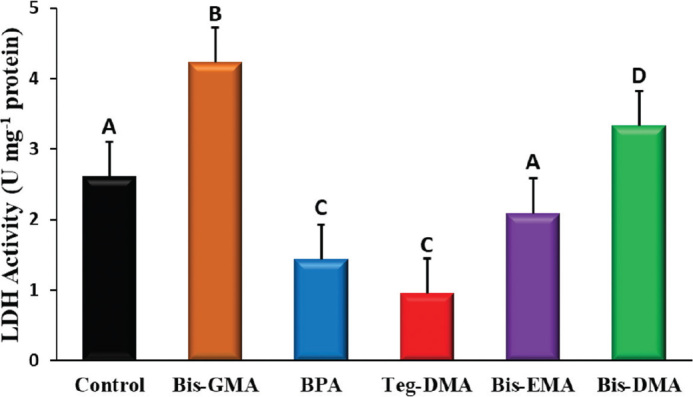
Effects of 24-h exposure to resin monomers on LDH activity (U mg^–^¹ protein) in A. salina nauplii. Data represent means ± SD. Means that do not share a letter are significantly different from each other (one-way ANOVA; Tukey’s pairwise comparisons).

Figure 8 shows the effects of the different monomers on protein -normalized LDH activity in *A. salina* nauplii. One way ANOVA revealed highly significant differences in LDH activity among different treatment groups (*F*₅,₁₈ = 77.46, *p* < 0.001). Tukey’s pairwise comparisons showed that Bis-GMA exerted the greatest activity, markedly higher than all the other monomers. *A. salina* nauplii exposed to Bis-DMA showed significantly higher LDH levels than the control and the Bis-EMA groups but did not reach the levels observed in the Bis-GMA-treated group. Conversely, nauplii exposed to BPA and TEG-DMA had the lowest LDH activities, both significantly lower than all other treatments, and not significantly different from each other. LDH activity did not differ significantly between the control and Bis-EMA-treated groups. Collectively the monomers exhibited a ranked response (Bis-GMA > Bis-DMA > Control ≈ Bis-EMA > BPA ≈ TEG-DMA), highlighting clear, compound-specific effects on LDH activity.

## Discussion

The present study evaluated the toxicity and developmental impacts of methacrylate monomers commonly used in resin-based materials, as well as BPA, on *A. salina*. The results showed distinct monomer and life-stage-specific effects on survival, post-hatch development, and membrane integrity. Although the concentrations tested (12.5–100 mg/L) exceed environmentally and clinically relevant exposure levels, they were intentionally selected for hazard screening and comparative material safety assessment rather than realistic exposure simulation. The monomers showed consistent effects on survival, development, and biochemical markers, with no opposing responses, suggesting compound-specific differences in toxicological responses rather than generalized stress effects.

The results of this study can be interpreted within a source–fate–exposure–effect framework (Figure 1), which helps place the findings in an ecological context. Dental monomers may enter aquatic systems from clinical sources via wastewater (source), where their distribution is shaped by processes such as dilution, persistence, and transformation (fate). Aquatic organisms like *A. salina* can then be exposed during early developmental stages through direct contact with dissolved compounds (exposure), resulting in the survival and developmental effects observed under laboratory conditions (effect). This framework helps structure and contextualize the biological responses reported in this study.

Of the monomers tested, BPA, Bis-GMA, and Bis-DMA caused progressively higher mortality with increasing dose, indicating genuine toxic effects rather than random mortality. In contrast, Bis-EMA was only mildly toxic, and TEG-DMA did not significantly affect survival at the concentrations examined. These findings suggest clear differences in toxic potential among monomers, as survival outcomes followed a consistent dose-related pattern, supported by estimated odds ratios.

For most monomers, naupliar body length proved to be a sensitive indicator of embryotoxicity, often revealing adverse effects at concentrations causing little or no mortality. BPA induced the strongest overall growth inhibition, causing reductions in body length ranging from ~9–53% following cyst exposure and ~3–19% following post-hatch exposure, with significant effects from ≥ 25 mg/L rather than a strictly linear dose–response pattern, in line with its endocrine-disrupting and developmental hazards. Bis-GMA showed moderate growth inhibition without a clear dose–response pattern, with reductions of approximately 13–19% following cyst exposure and variable effects post-hatch, reflecting limited separation among treatment groups.

In contrast, Bis-DMA and Bis-EMA both showed limited developmental effects, with Bis-DMA displaying inconsistent and weak concentration-dependent changes, while Bis-EMA showed clearer reductions in naupliar body length at higher concentrations (50 and 100 mg/L). Neither compound showed effects after cyst-stage exposure. TEG-DMA affected growth in a stage-dependent and nonmonotonic manner. During naupliar exposure, body length was significantly reduced at 12.5 and 25 mg/L, while no significant effects were observed at 50 or 100 mg/L, indicating a non-dose-dependent response; overall reductions ranged from ≈9–13%. In contrast, cyst-stage exposure resulted in a stronger overall reduction in body length (≈22–35%) although the response did not follow a strictly linear concentration–effect pattern across doses.

Overall, the patterns varied by compound, with clear dose-related effects for BPA, consistent inhibition without strict dose-dependence for TEG-DMA, moderate effects for Bis-GMA with limited dose–response structure, and weak or nonsignificant responses for Bis-DMA and Bis-EMA, indicating compound-specific rather than uniform concentration–response relationships.

It is important to note that developmental effects occurred even at concentrations that did not affect survival. This highlights the importance of sub-lethal endpoints in toxicity profiling, as evidenced by the dissociation between survival and naupliar development, particularly for TEG-DMA [22]. The observed decreases in body size, although variable across compounds, may affect feeding, swimming capacity, and overall fitness, although these observations reflect potential biological hazard under experimental conditions rather than direct environmental risk, with potential implications for population viability.

Early developmental exposure during the cyst stage generally enhanced the observed effects, with hatched nauplii showing more substantial decreases in body size relative to post-hatch-exposed counterparts. This effect was most pronounced for BPA and TEG-DMA and to a lesser extent for Bis-GMA and Bis-EMA. Although Bis-EMA showed greater percentage reductions following cyst-stage exposure, these effects were not statistically significant. In contrast, both Bis-DMA and Bis-EMA showed statistically significant reductions during naupliar exposure, indicating a deviation from the overall stage-dependent trend. Cyst-stage exposure resulted in marked body length reductions (9–53% for BPA, 23–26% for TEG-DMA, 13–19% for Bis-GMA, and 9–12% for Bis-EMA), whereas post-hatch exposure produced comparatively smaller effects (3–19% for BPA, 9–13% for TEG-DMA, −1 to 12% for Bis-GMA, and 2–8% for Bis-EMA, with significance observed only during naupliar exposure for Bis-DMA and Bis-EMA). Collectively, these results indicate heightened vulnerability during early development and a greater impact of early life exposure on developmental toxicity, in agreement with previous reports in aquatic ecotoxicology [23].

Differences in lipid affinity among resin-based monomers may influence their biological interactions and internal exposure. Based on calculated partition coefficients, Bis-GMA is among the most hydrophobic monomers, while BPA has intermediate hydrophobicity, and TEG-DMA shows lower hydrophobicity due to its glycol-based backbone [24, 25]. For Bis-DMA and Bis-EMA, direct measurements of log P are not readily available; therefore, hydrophobicity is commonly inferred from molecular structure or physicochemical properties. On this basis, a widely accepted qualitative ranking for hydrophobicity is Bis-GMA > Bis-DMA ≈ Bis-EMA > BPA > TEG-DMA. Comparison with this ranking suggests only partial correspondence between lipid affinity and biological effects. Although more hydrophobic monomers such as Bis-GMA and Bis-DMA were associated with reduced survival and growth, BPA, despite intermediate hydrophobicity, also produced marked effects, and TEG-DMA caused substantial growth inhibition in the absence of mortality. Collectively, these findings indicate that hydrophobicity provides useful context but does not fully explain the observed toxicity patterns.

The differences in developmental toxicity observed among the tested monomers indicate compound-specific effects rather than a single governing mechanism. LDH activity, a widely used biomarker of metabolic disturbance and cellular stress [26–28], was elevated following Bis-GMA and Bis-DMA exposure, consistent with compromised membrane integrity and cytotoxic injury [26–28]. Notably, Bis-DMA increased LDH activity despite showing no significant effect on naupliar growth, indicating a dissociation between cellular stress and morphological outcomes. Although hydrophobicity may influence cellular uptake, it did not consistently predict membrane damage: Bis-EMA did not significantly increase LDH activity despite its relatively high hydrophobicity, whereas Bis-GMA did.

Although LDH activity was used as a general indicator of cellular integrity, its nonspecific nature limits direct mechanistic interpretation. In the present study, significant reductions in LDH activity were observed in nauplii exposed to BPA and TEG-DMA, despite clear growth inhibition (particularly for BPA and TEG-DMA) and effects on survival for BPA. These findings indicate that growth and survival inhibition are not necessarily associated with increased LDH release and therefore may not reflect overt membrane damage as measured by this assay.

LDH responses were compound specific, with Bis-GMA and Bis-DMA showing increased activity, whereas BPA and TEG-DMA showed reductions, and only some of these changes corresponded to growth effects. While these patterns suggest differential cellular responses among monomers, LDH alone is insufficient to infer underlying biological mechanisms. Therefore, potential processes such as membrane disruption, oxidative stress, endocrine disruption, or metabolic perturbation remain speculative and require further targeted investigation [29, 30].

The absence of significant LDH changes following Bis-EMA exposure suggests limited effects on cellular membrane integrity, although conclusions based on this marker should be made cautiously given its nonspecific nature. Overall, LDH responses should be interpreted as indicators of general cellular disturbance rather than specific pathways, highlighting the importance of integrating cellular biomarkers with growth endpoints to detect early or subtle toxic effects.

It is also important to consider methodological limitations related to exposure characterization. Analytical verification of exposure concentrations (including stability, solubility, and bioavailability) was not performed; therefore, nominal concentrations were used. Although fresh solutions were prepared for each experiment to minimize degradation, this remains a limitation of the study, particularly for hydrophobic compounds where adsorption or partitioning may occur. As a result, discrepancies between nominal and actual exposure concentrations may exist, especially for hydrophobic monomers such as Bis-GMA due to adsorption to surfaces or partitioning effects.

The concentrations used in this study (12.5–100 mg/L) are several orders of magnitude higher than those reported in environmental systems, which are typically in the ng/L to µg/L range. These exposures therefore represent hazard-screening conditions and should not be directly extrapolated to real- world environmental or ecological risk scenarios. Standard ecotoxicological endpoints such as Lethal Concentration 50% (LC50) or No Observed Effect Concentration (NOEC) were not determined in this study because the experimental design used a limited number of discrete concentrations rather than a full dose–response curve suitable for threshold modeling. In addition, such parameters are not currently available in the literature for these monomers in comparable biological systems, which further limits direct comparison.

Nevertheless, the observed patterns of response (rather than consistent concentration-dependent trends across all compounds) provide useful information for hazard screening and for comparing the relative toxicity of the tested monomers within this study. Future work using refined dose–response designs would be needed to establish standard ecotoxicological benchmarks and improve environmental comparability. Additionally, studies employing environmentally relevant concentrations and a broader range of biological and biochemical endpoints would help provide a more comprehensive understanding of the ecological effects of these monomers.

Finally, it is important to note that these findings reflect hazard under controlled experimental conditions rather than environmental risk. While the relatively high concentrations used in this study revealed toxic effects, environmental exposures are expected to be lower. Therefore, these findings indicate potential hazard rather than direct environmental risk. Long-term and environmentally realistic exposure studies are still needed to better assess ecological safety and support the development of greener materials.

## Conclusion

Collectively, these findings demonstrate differences in the toxic potential of resin-based monomers, with growth-related developmental endpoints being more sensitive than survival alone. The amplified responses observed following cyst-stage exposure underscore the importance of including early life-stage endpoints in hazard assessment. Although the concentrations tested exceed environmentally and clinically relevant levels, these results provide a comparative basis for monomer toxicity and highlight the limitations of relying solely on acute mortality to estimate ecological effects associated with resin materials. Overall, these findings indicate potential biological effects under hazard-screening conditions, and additional studies at environmentally relevant exposure levels are needed to better evaluate ecological safety. Importantly, the effects varied between compounds and were not consistently dose-dependent across all monomers, so these patterns should be interpreted with appropriate caution when drawing broader conclusions.

## Data Availability

The data presented in this study are available on request from the corresponding author.
